# Comparable response of *ccn1 *with *ccn2 *genes upon arthritis: An *in vitro *evaluation with a human chondrocytic cell line stimulated by a set of cytokines

**DOI:** 10.1186/1478-811X-3-6

**Published:** 2005-04-15

**Authors:** Norifumi H Moritani, Satoshi Kubota, Toshio Sugahara, Masaharu Takigawa

**Affiliations:** 1 Department of Biochemistry and Molecular Dentistry, Okayama University Graduate School of Medicine and Dentistry, Okayama 700-8525, Japan; 2 Department of Oral and Maxillofacial Reconstructive Surgery, Okayama University Graduate School of Medicine and Dentistry, Okayama, Japan

## Abstract

**Background:**

The chondrosarcoma-derived HCS-2/8 has been known to be an excellent model of human articular chondrocytes. By mimicking the arthritic conditions through the treatment of HCS-2/8 cells with cytokines, we estimated the gene expression response of ccn1 and ccn2 during the course of joint inflammation *in vitro*.

**Results:**

In order to mimic the initiation of inflammation, HCS-2/8 cells were treated with tumor necrosis factor (TNF)-α. To induce pro-inflammatory or reparative responses, TGF-β was employed. Effects of an anti-inflammatory glucocorticoid were also evaluated. After stimulation, expression levels of *ccn1 *and *ccn2 *were quantitatively analyzed. Surprisingly, not only *ccn2*, but also *ccn1 *expression was repressed upon TNF-α stimulation, whereas both mRNAs were uniformly induced by transforming growth factor (TGF)-β and a glucocorticoid.

**Conclusion:**

These results describing the same response during the course of inflammation suggest similar and co-operative roles of these 2 ccn family members in the course of arthritis.

## Background

Osteoarthritis (OA) and rheumatoid arthritis (RA) are most common orthopaedic complexities among aged individuals [1]. Although they may be etiologically distinct each other, both are characterized by progressive destruction of articular cartilage, which is mainly caused by inflammatory stresses [1]. The most major problem in the therapeutics of these joint diseases is the difficulty in regenerating damaged articular cartilage. It is widely recognized that even a defect of 2 mm in diameter in normal articular cartilage may not be repaired naturally [2,3]. Therefore, cartilage regeneration is one of the most preferred subjects for the investigators in medical research field.

In our previous report, we for the first time clarified that CCN2/connective tissue growth factor (CTGF) was capable of regenerating full-thickness defect in articular cartilage and promoted the repair of damaged cartilage in an OA model *in vivo *[2]. Also, expression of *ccn2 *in OA cases was reported [3]. As such, it is now quite clear that CCN2 is involved in inflammatory response and repair process of articular cartilage during arthritis. Therefore, it is of critical importance to investigate the regulation of *ccn2 *expression along with the course of joint inflammation.

The CCN family is a novel group of proteins that act as multiple mediators among a variety of extracellular signaling molecules. This family of secreted proteins consists of 6 members: Cyr61 (CCN1), CTGF (CCN2), Nov (CCN3), Elm-1/WISP-1 (CCN4), rCop-1/WISP-2/CTGF-L (CCN5), and WISP-3 (CCN6) [4-9]. These structurally conserved proteins share 4 modules with sequence similarities to insulin-like growth factor-binding proteins, von Willebrand factor type C module, thrombospondin type 1 repeat, and growth factor cysteine knots, respectively [4-9]. CCN2/CTGF is a classical member of CCN family and shares significantly functional similarities with another member, CCN1/Cyr61. Both proteins share –45% amino acid sequence identity [10], bind heparin, are associated with the ECM [11], and exhibit remarkable functional versatility [12,13]. CCN1 and CCN2 can stimulate chemotaxis and promote proliferation of endothelial cells and fibroblasts in culture, induce neovascularization *in vivo*, and promote chondrogenic differentiation, the last-mentioned action being consistent with their expression in prechondrogenic mesenchyme during embryogenesis [5-7,13-15]. Considering such biological involvement of CCN1 in mesenchymal tissue formation, active roles of CCN1 in cartilage regeneration and repair may not be disregarded as well.

Therefore, in our present study, we assessed the gene regulation profile of CCN1 as well as CCN2 in a human chondrocytic cell line, HCS-2/8, upon the stimulation mimicking the course of chronic inflammation. This particular cell line was selected, since HCS-2/8 was established from a chondrosarcoma and has best retained a variety of mature chondrocytic phenotypes [16-18]. Using this *in vitro *model, we found CCN1 may also be involved in the inflammatory response in joints and may be useful in cartilage regenerative therapeutics, as described in CCN2.

## Results

### Strict and discriminating quantification of *ccn1 *and *ccn2 *mRNAs by real-time-RT-PCR

**Figure 1:**
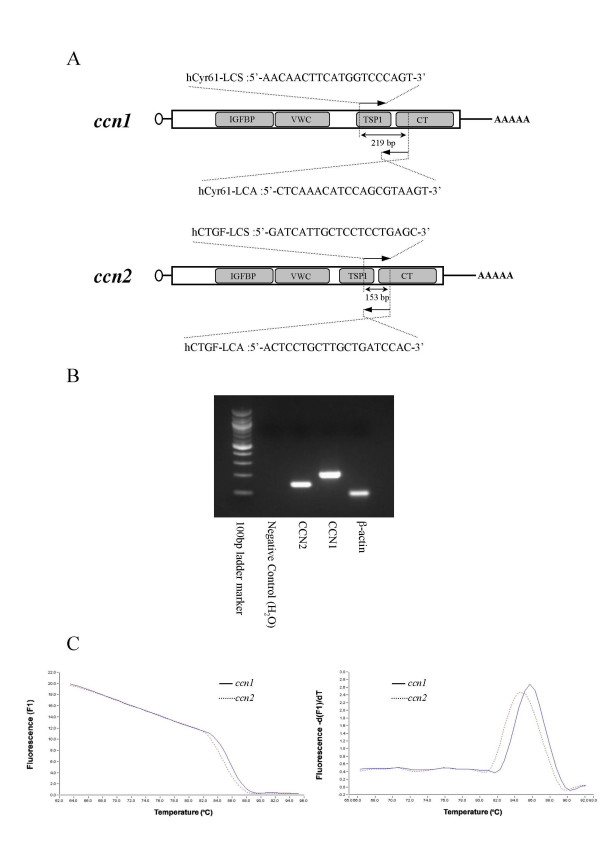
**Distinctive quantification of ccn1 and ccn2 mRNA by real-time RT-PCR**. **A. **Primers used for real-time PCR and the structures of human *ccn1 *and *ccn2 *mRNAs. Schematic representations are illustrated in reference to the modular structure of human CCN1 and CCN2 (stippled boxes). The small open circle and AAAAA at the left and right ends denote the 5'-cap structure and poly-A tail, respectively. Names, locations for recognition, nucleotide sequence of the primers, and the expected sizes of the PCR products are given. Abbreviations: IGFBP, insulin-like growth factor binding module; VWC, von Willebrand factor type C module; TSP1, thrombospondin type 1 repeat; CT, C-terminal module. **B. **The CCN1 (219 bp), CCN2 (153 bp), and β-actin (101 bp) PCR products amplified by LightCycler were analyzed by agarose electrophoresis. **C. **Melting curve analysis of the RT- PCR products of *ccn1 *and *ccn2*. Melting curves were acquired after 45 cycles of amplification. Melting curve pattern is displayed on the left panel, where fluorescent intensity (F1) from SYBR green is plotted against temperature. Melting peak pattern can be found on the right panel, in which the decrement of F1 is plotted against temperature.

Since *ccn1 *and *ccn2 *are members of the same gene family, it is critically important to examine the specificity of each quantification system in order to avoid possible cross-recognition [4]. First, RT-PCR products of the initial amplification under our protocol were separated by 2 % agarose gel electrophoresis. All of the amplified LightCycler-PCR products showed single bands of the expected lengths (i.e., 219 bp for CCN1, 153 bp for CCN2, and 101 bp for β-actin; Fig. 1B). Second, we further verified the identity of each PCR product by direct nucleotide sequencing. As a result, sequence analysis of the PCR products showed 100 % homology to the published sequences. After these initial examinations, specificity of all of the products was confirmed each time by melting curve analysis *via *LightCycler Software 3.39 (Roche). Melting curves were analyzed for all PCR runs. Continuous fluorescence monitoring while slowly elevating the temperature resulted in a sudden decrease in SYBR green I (Roche) fluorescence intensity, when denaturation of the PCR product occurred. Thus, the melting curve analysis revealed a specific pattern for each target. Figure 1C shows an example of a melting curve analysis, indicating no nonspecific PCR product. As such, accurate and distinctive quantification of *ccn1 *and *ccn2 *was deemed warranted.

### Repressive response of both *ccn1 *and *ccn2 *genes upon inflammatory provocation by TNF-α

**Figure 2:**
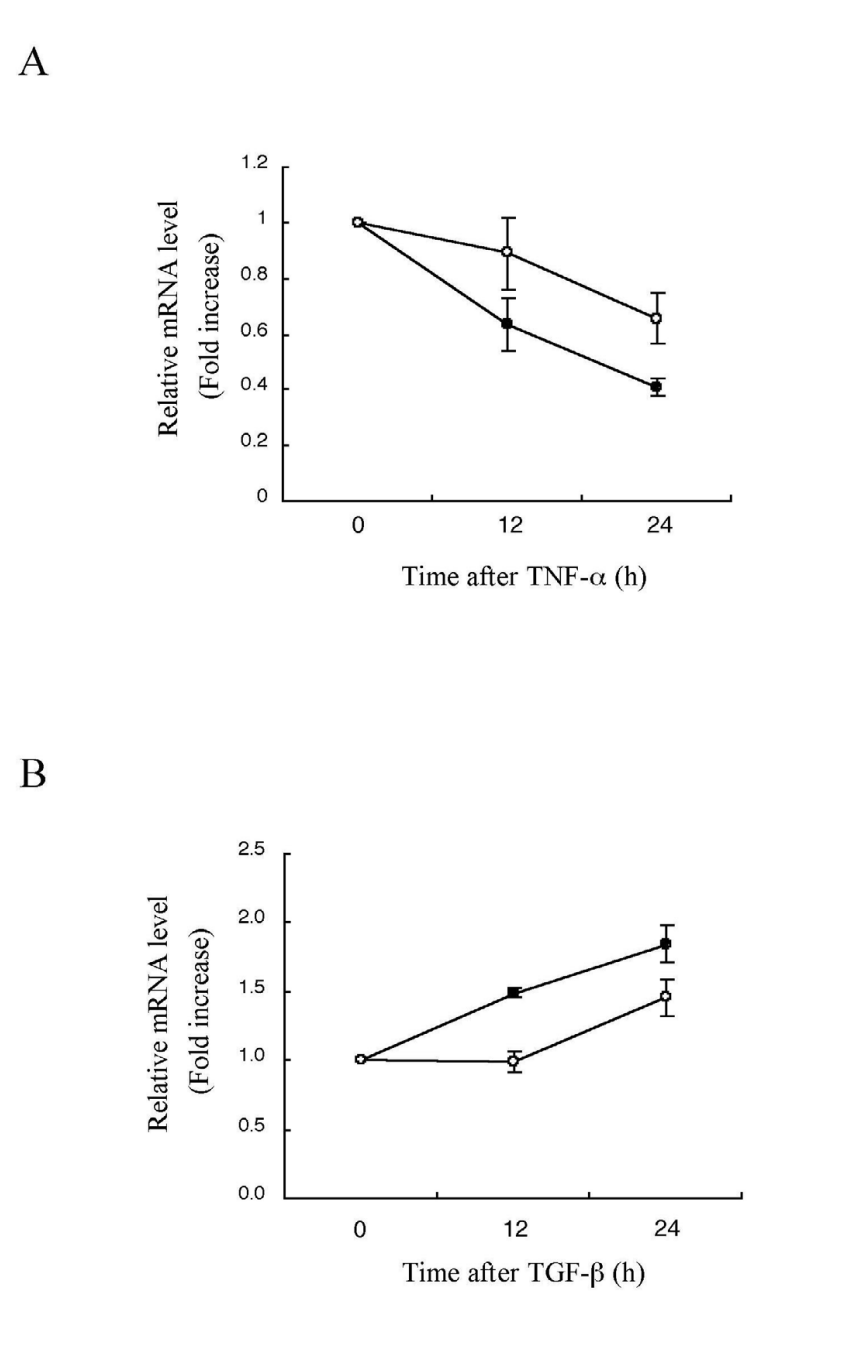
**Coordinative repression and induction of *ccn1 *and *ccn2 *transcripts in response to inflammatory TNF-α (A) and pro-inflammatory TGF-β (B) in chondrocytic HCS-2/8 cells**. Results of mRNA quantification by real-time RT-PCR analysis are displayed. Plotted values represent relative mRNA copy numbers versus those at time 0. Each copy number was computed by standardizing the raw data against those of β-actin. Open circles, *ccn1*; solid circles, *ccn2*. Mean values and standard deviations (SDs) of at least 2 experiments are shown.

TNF-α is one of the best-known inflammatory cytokines and is involved in a number of inflammatory diseases including arthritis [1]. Therefore, we evaluated the effect of TNF-α on the expression of the *ccn1 *and *ccn2 *genes in the chondrocytic HCS-2/8 cells. HCS-2/8 cells were treated with 10 nM TNF-α for 12 – 24 hours. By this stimulation, *ccn2 *mRNA levels were repressed 0.6 (0.64) to 0.4 (0.41) fold, and *ccn1 *mRNA levels, 0.9 (0.89) to 0.7 (0.66) fold, by 24 h (Fig. 2A). The result that both genes were uniformly downregulated upon TNF-α stimulation strongly suggests similar or complementary functions of these gene products in the middle of inflammatory process.

### Induction of *ccn1 *and *ccn2 *by pro-inflammatory and regenerative signal in chondrocytic HCS-2/8 cells

Tissue repair and regeneration are the last step of inflammation, in which damaged tissue is initially filled with fibroblasts surrounded by a vast amount of extracellular matrices (ECM). However, in general, inflammatory stimulation and ECM formation occur in parallel along the course of chronic inflammation. Through such process, TGF-β and CCN2 is known to critically regulate ECM deposition from a variety of mesenchymal cells. Molecular regulation of *ccn2 *by TGF-β has been relatively investigated [19,20]. Nevertheless, little is known concerning the interplay between *ccn1 *and TGF-β in cartilage. As such, we evaluated the response of *ccn1 *gene in chondrocytic HCS-2/8 cells to TGF-β, in comparison with *ccn2*. Treatment with 10 ng/ml TGF-β induced *ccn2 *mRNA up to 1.5 to 1.9 fold; and *ccn1 *mRNA, by 1.5 fold up to 24 h (Fig. 2B). Additionally, we found that these mRNAs were better induced by 50 ng/ml TGF-β than by 10 ng/ml (data not shown). These findings suggest coordinative functions of *ccn1 *and *ccn2 *in repair and chronic inflammation of articular cartilage.

### Anti-inflammatory treatment and expression of *ccn1 *and *ccn2 *genes

**Figure 3:**
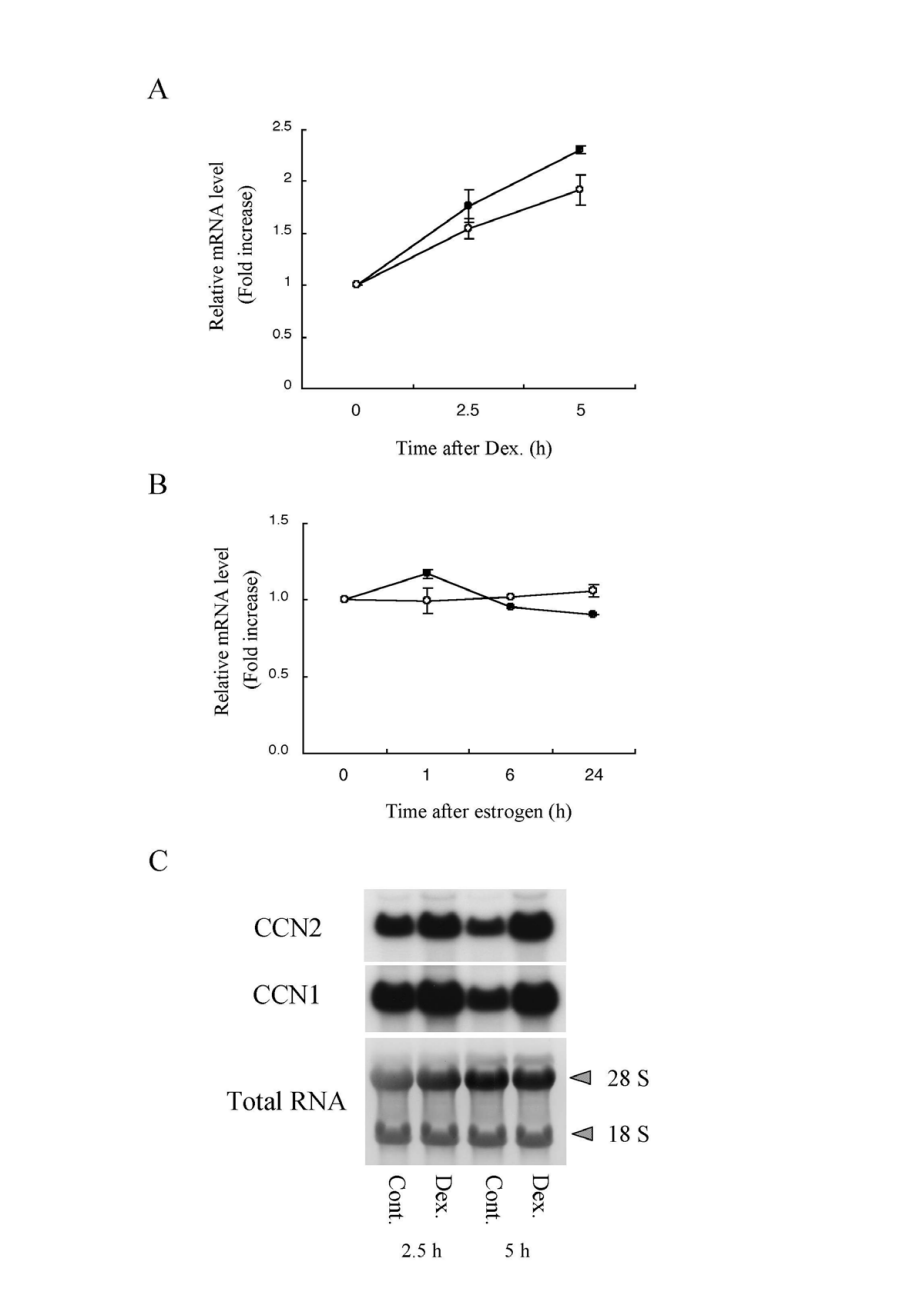
**Comparable response of *ccn1 *and *ccn2 *to anti-inflammatory dexamethasone in HCS-2/8 cells**. **A. **Expression levels of *ccn1 *and *ccn2 *transcripts examined by quantitative real-time PCR. Plotted values represent relative mRNA copy numbers, as described in the legend to Figure 2. Open and solid circles denote *ccn1 *and *ccn2*, respectively. **B. **Control experiments with estrogen, showing no significant alteration in gene expression either in *ccn1*, or *ccn2*. These data are mean values of 2 experimental results shown with error bars (SD). **C. **Northern blot analysis of *ccn1 *and *ccn2 *gene expression in HCS-2/8 cells in response to dexamethasone treatment. The *ccn1 *and *ccn2 *mRNAs (upper and middle panels) and total RNAs stained with 0.02 % methylene blue (lower panel) are shown. : 28S, 28S ribosomal RNA; 18S, 18S ribosomal RNA.

Glucocorticoids are known to possess strong anti-inflammatory effects and also to be involved in the control of cartilage metabolism [21]. Particularly, the effectiveness of glucocorticoids on RA symptom is so prominent that it is frequently applied clinically, despite its adverse systemic effects. It is already known that *ccn2 *gene expression is induced by glucocorticoids in chondrocytic cells as well as in fibroblasts [22,23]. In contrast, molecular interaction between glucocorticoids and *ccn1 *gene in chondrocytes has not been investigated. Thus, we performed Northern blot analysis, as well as the real-time RT-PCR quantification to estimate the effects of glucocorticoids on *ccn1 *and *ccn2 *gene expression. When HCS-2/8 cells were treated with 50 nM dexamethasone for 2.5 – 5 hours and then examined by real-time PCR, *ccn2 *mRNA was induced by 1.8 to 2.3 fold, and *ccn1 *mRNA was similarly induced by 1.5 to 1.9 fold up to 5 h (Fig. 3A). The results obtained by Northern blot analysis were quite similar to the PCR ones (Fig 3B).

As a control experiment, we carried out similar analysis with 17β-estradiol, which is another steroid hormone and is also one of the regulators to maintain the homeostasis of connective tissue. However, treatment of HCS-2/8 cells with 10 nM estrogen for 1 – 24 hours resulted in no significant change in *ccn1 *and *ccn2 *mRNA expression levels. In fact, no effects were observed even up to the concentration of 100 nM (data not shown).

## Discussion

In this study, we comparatively analyzed the expression profiles of *ccn1 *and *ccn2*, while simulating the course of arthritis; i.e., inflammation, tissue regeneration and anti-inflammatory treatment, utilizing a human chondrocytic HCS-2/8. In advance to the evaluation, we established a real-time PCR quantification method by using a LightCycler system. In view of the data provided for sensitivity, linearity, and reproducibility, this assay system accurately allowed the discriminating quantification of these mRNAs from the same gene family. The total reliability of this system was evident, as represented by the facts that every specific primer set produced distinct and specific PCR products (Fig. 1) and quantitative results were comparable to those of Northern blot analysis (Fig. 3).

The involvement of CCN2 in arthritic diseases has been indicated. In fact, expression of *ccn2 *gene in clinical cases was reported [3]. Also in an experimental OA model, distinct induction of *ccn2 *gene expression was observed. These findings are regarded as a regenerative response of damaged cartilage, since exogenously applied CCN2 was proven to be effective in cartilage regeneration. In rat models, CCN2 captured in collagen hydrogel to allow gradual release of this factor efficiently promoted the regeneration of full-thickness cartilage defect and experimentally induced OA cartilage [2]. Therefore, expression profile of *ccn2 *in chondrocytes along the time course of inflammation is thought to represent the proper gene regulation to provide a regenerative molecule during arthritis, and thus itself is worth investigated. More interestingly, *ccn1 *gene expression exactly followed the fluctuation pattern of *ccn2* gene expression upon any kind of stimulation tested. These results clearly indicate that not only CCN2, but also CCN1 may be provided as a regenerative molecule in arthritis. This hypothesis is supported by a number of their functional similarities. Indeed, these factors are associated with the ECM, stimulate chemotaxis and promote proliferation of endothelial cells and fibroblasts, and promote neovascularization and chondrogenic differentiation [7-15,24]. Considering such similarities and the concomitant fluctuation of gene expression upon inflammation together, CCN1 is expected to be one of the useful molecular tools to promote cartilage regeneration. In order to examine this hypothesis, it is necessary to evaluate the regenerative potential of CCN1 protein in damaged articular cartilage, as was examined with CCN2. *In vivo *evaluation of the expression of *ccn1 *upon OA and RA and the effects of CCN1 protein on cartilage regeneration is currently in progress. Since all of the CCN family members are thought to be mediators of multiple signaling molecules, therapeutic utility of another member, such as CCN3/NOV is also expected and obviously, need to be explored.

## Conclusion

*In vitro *simulation of arthritis with a human chondrocytic cell line revealed the same response pattern of *ccn1 *as that of *ccn2*, which is known as a regenerative mediator in cartilage repair. Together with similar functionalities of CCN1 and CCN2 in mesenchymal tissues, these results suggest possible utility of CCN1 in regenerative therapy of damaged mesenchymal tissues.

## Methods

### Materials

TNF-α and TGF-β1 were purchased from Promega (Madison, WI, USA). Dexamethasone and estrogen (17β-estradiol) were purchased from Sigma (St. Louis, MO,USA).

### Cell culture

HCS-2/8 cells, a chondrocytic cell line derived from a well-differentiated type of human chondrosarcoma [19], were maintained in Dulbecco's modified Eagle's medium (D-MEM) supplemented with 10 % fetal bovine serum (FBS) under an atmosphere of humidified air containing 5 % CO_2_. In the experiments in which the effect of estrogen was studied, the medium was replaced with phenol red-free DMEM and 2 mM glutamine (Nissui Pharmaceutical Co. Ltd., Tokyo, Japan) containing 2 % charcoal-treated FBS, after the HCS-2/8 cells had become subconfluent. In the experiments with TGF-β1, dexamethasone, and estrogen, the cells were harvested after the treatment of subconfluent cells (simulating growing phase upon regeneration) with each factor for the desired time periods. In the TNF-α experiment, the cells were harvested after the treatment of confluent cells (simulating quiescent phase before inflammatory damage) with the factor for the desired time periods.

### RNA extraction and reverse transcription (RT)

Total RNA was extracted from HCS-2/8 cells by the acid guanidinium phenol-chloroform method previously described [25]. Reverse transcription by avian myelosarcoma virus reverse transcriptase was carried out by using a commercially available kit (Takara Shuzo, Tokyo, Japan) and 1.0 μg of total RNA. Then, the samples were diluted by 20-fold with RNase-free H_2_O for subsequent quantification.

### Quantitative real-time PCR amplification

On the basis of the published cDNA sequences of CCN2/CTGF (GenBank accession no. NM_001901) and CCN1/Cyr61 (no. AF031385), specific primers were designed for each. Their nucleotide sequences are displayed in Fig. 1. Real-time quantitative PCR was performed with a LightCycler system (Roche Molecular Biochemicals, Mannheim, Germany). For each assay, reaction mixtures containing 2.0 μl of a cDNA pool, 1.0 μl of LC DNA Master SYBR Green I mixture (Roche), 50 ng of the primers, and 0.8 μl of 25 mM MgCl_2 _were prepared on ice. After the reaction mixtures had been loaded into glass capillary tubes, amplification was performed under the following cycling conditions: initial denaturation at 95°C for 10 min, followed by 45 cycles of denaturation at 95°C for 15 sec, annealing at 55°C for 10 sec, and extension at 72°C for 10 sec. The temperature transition rate was set at 20°C/ sec. The fluorescence representing double-strand DNA formation was measured in single-acquisition mode at 72°C after each cycle. For each sample, the cDNA copy numbers of the target and an internal control (β-actin) genes were determined based on calibration curves (see below). The relative amount of the target cDNA was then computed by dividing the copy number by that of the internal control to obtain a normalized value. Separate calibration curves for *ccn1*, *ccn2*, and β-*actin *were prepared with serially diluted plasmid DNAs containing the target sequences, which were amplified and evaluated simultaneously in each assay. To distinguish specific signal from non-specific products, melting curve analysis was performed after each amplification cycle. Samples were maintained at 63°C for 10 sec, and then the temperature was gradually increased to 95°C at a rate of 0.1°C/sec, while the signals were monitored with a step-acquisition mode, as described previously [26]. The real-time PCR analysis condition was optimised to a level without non-specific amplification under an acceptable PCR efficiency (a slope ranging from -2.9 to -4.5). To verify the melting curve results, we analyzed representative PCR samples in 2.0 % agarose gels, purified and directly sequenced them from both directions by an automated DNA sequencer (ABI prism 310 Genetic Analyzer; Applied Biosystems, Foster City, CA, USA).

### Northern blot analysis

Ten micrograms of total RNA was electrophoresed in formaldehyde agarose gel and subsequently blotted onto a nylon membrane. For hybridization probes, CCN2/CTGF and CCN1/Cyr61 cDNA fragments were prepared by RT-PCR with pairs of specific primers. Primers specific for CCN2/CTGF [27] and CCN1/Cyr61 [28] were described previously. These PCR products were radiolabeled and used for hybridization as described earlier [20].

## Competing interests

The author(s) declare that they have no competing interests.

## Authors' contributions

NHM performed molecular biological studies and prepared most part of data. SK arranged the constitution of the entire work and drafted the manuscript. TS participated in the design of the work. MT participated in coordination and drafting the manuscript and provided general support.
